# Serum metabolites with diagnostic potential in prostate cancer and the inhibitory effects of alpha-Tocomonoenol on prostate cancer cells

**DOI:** 10.3389/fonc.2025.1691767

**Published:** 2025-11-20

**Authors:** Yang Shen, Bo Liu, Yuhua Zhou, Jing Lv, Xiang Zhang, Chun Yang, Yuwei Zhang, Ninghan Feng

**Affiliations:** 1 Wuxi Medical Center, Nanjing Medical University, Wuxi, China; 2 Department of Urology, Jiangnan University Medical Center, Wuxi, China; 3 Wuxi No.2 People's Hospital, Medical School of Nantong University, Nantong, China; 4 Wuxi School of Medicine, Jiangnan University, Wuxi, China

**Keywords:** prostate cancer, PSA gray zone, early detection, serum metabolites, alpha-tocomonoenol

## Abstract

**Introduction:**

Prostate cancer is the most common malignant disease of the male urinary system, seriously endangering men’s health. Currently, early detection of prostate cancer mainly relies on prostate-specific antigen (PSA) screening; however, PSA is characterized by high sensitivity but low specificity. Patients with PSA levels in the gray zone (4-10ng/ml) are at risk of excessive medical interventions, highlighting the great significance of exploring new diagnostic indicators for prostate cancer.

**Methods:**

A total of 60 subjects were enrolled in this study, including 30 prostate cancer patients and 30 benign prostatic hyperplasia patients. Non-targeted metabolomics analysis was performed on the subjects, and the profiling results were statistically analyzed. A diagnostic model was constructed using stepwise regression, and experimental verification was conducted on alpha-Tocomonoenol, one of the key differential metabolites. Additionally, the effect of alpha-Tocomonoenol on cell proliferation was evaluated in LNCaP, 22Rv1, and RWPE-1 cells.

**Results:**

Six differential metabolites between the prostate cancer group and the benign prostatic hyperplasia group were used to construct a diagnostic model, which showed a high area under the curve (AUC = 0.9433). Experimental verification revealed that alpha-Tocomonoenol inhibited the proliferation of LNCaP and 22Rv1 cells by binding to androgen receptors, while it had no significant effect on the proliferation of RWPE-1 cells.

**Discussion:**

This study demonstrates that serum metabolites have the potential to serve as diagnostic biomarkers for the early detection of prostate cancer. Furthermore, alpha-Tocomonoenol might exert an anti-proliferative effect on prostate cancer cells through binding to androgen receptors, providing new insights into the development of novel diagnostic tools and therapeutic strategies for prostate cancer.

## 1 Introduction

Prostate cancer (PCa), as the second most common cancer among men, has a high incidence and mortality worldwide. In 2020, over one million new cases of PCa were diagnosed globally, resulting in more than 300, 000 fatalities across world (1). In 2022, prostate cancer accounted for more than 1.4 million new cases and nearly 400, 000 deaths worldwide (2). PCa is still a major health problem for men up to now. The early detection along with timely intervention of PCa hold great significance for patients. PCa early detection can protect patients from advanced or metastatic stages, significantly enhance patients’ quality of life, and effectively lower the mortality rate (3, 4). PCa screening methods include multi-parameter MRI, and some prostate-specific antigen (PSA)-based auxiliary markers, such as prostate health index (PHI) and kallikrematory protein HK2, among which PSA screening is the most widely used approach (5, 6).

PSA, a protein specifically secreted by prostate epithelial cells, has been used in PCa screening and early diagnosis for more than 30 years. Detection of serum PSA level combined with rectal palpation has greatly improved the diagnostic accuracy of early PCa (7). Although PSA is currently recognized as a vital screening measure for PCa around the world, it still has important limitations. Besides PCa, elevated serum PSA levels can also be observed in various benign conditions, including prostatitis and benign prostatic hyperplasia (BPH). In other words, PSA is highly sensitive but not specific for PCa detection. PCa screening using PSA carries a high risk of false positives, especially the patients with serum PSA gray zone level of 4-10ng/ml (8). For this patient cohort requiring long-term monitoring, frequent hospital visits impose substantial logistical and psychological burdens. Therefore, the development of non-invasive diagnostic biomarkers enabling home-based detection of PCa progression could revolutionize disease management while enhancing patient compliance.

Metabolites are the direct products of cell metabolism and can quickly reflect the physiological and pathological state of the organism, they also have the potential to enable recording of early biochemical changes in disease (9). The different stages of PCa always have unique metabolic features, and some metabolism has shown to be essential for multiple biological processes in PCa (10, 11). The metabolomics as a systematic analysis, is able to reflect a comprehensive profile of disease status (12). Therefore, metabolism looks like an exceptional opportunity to develop excellent biomarkers for diagnosis of PCa. Using serum differential metabolites, we expect to construct a diagnostic model to assist in the diagnosis of early prostate cancer.

alpha-Tocomonoenol (A-T) is a form of vitamin E (VE) in animals and plants (13). Because of the essential antioxidant functions of VE, it is thought that it may also have the cancer prevention activity (14). However, in a large cohort study of vitamin E and prostate cancer risk, the researchers found that healthy men with average risk of prostate cancer subjected to vitamin E (400 IU per day) have a significantly increased risk of prostate cancer (15). A-T may have the potential to be a biomarker for prostate cancer.

## 2 Materials and methods

### 2.1 Sample collection and processing

This study was approved by the Ethics Committee of Jiangnan University Medical Center (2023-Y-168) and been retrospectively registered in the ISRCTN registry with TRN ISRCTN14665000 and registration date 17 December 2024. Informed consent was obtained from all subjects and/or their legal guardian(s). Initial sample collection involved 220 hospitalized patients at department of urology, Jiangnan University Medical Center from November 2023 to June 2024, with final enrollment of 60 subjects determined through rigorous application of inclusion/exclusion criteria and pathological assessment. All blood samples were collected before treatment. And all samples were processed within 1 hour. In detail, the blood samples were centrifuged at 4000rpm for 10min at 4°C, and 1ml of upper serum of each sample was stored at -80°C in the sterile EP tube.

All samples were further screened and grouped into the BPH group and the PCa group based on their pathological results. Specifically, here are the exclusion criteria we considered:

1. Patients with retested PSA values outside 4-10ng/ml range after admission were excluded.
2. Patients who had received treatment for prostate-related diseases in the past 3 months were excluded.
3. Patients whose blood samples were non-fasting were excluded.
4. Patients with other tumors were excluded.

### 2.2 Serum metabolite profiling

All serum samples were sent to Hangzhou Lianchuan Biological Information Co., Ltd. for non-targeted metabolite profiling. Specifically, we used organic reagent precipitation metabolites method to extract metabolites from serum samples. All metabolites were randomly sorted for analysis, including quantitative analysis, correlation analysis, and differential analysis. In addition, KEGG functional enrichment analysis, and interaction network analysis of differential metabolites were performed. All results were quality controlled and reproducibly evaluated.

#### 2.2.1 Details of serum metabolite profiling methods

In our study, the analytical platform of serum metabolite profiling was LC–MS, and the Ionization modes were positive- and negative-ion modes. The details of Data-preprocessing pipeline were that peak picking, peak grouping, retention-time correction, second-round peak grouping, and annotation of isotopes and adducts were performed with XCMS. Subsequent data processing and analyses were carried out using the R-based toolbox comprising XCMS, CAMERA, and metaX. We adopted the medium normalization. And our quality-control strategy was that Extracted samples were randomized prior to injection. QC aliquots were inserted at the beginning, middle, and end of the run sequence to serve as technical replicates. Technical reproducibility was evaluated by computing Pearson’s correlation coefficients between the log-transformed abundance profiles of all QC samples.

The above content is from the “Non-Target Metabolomics Experiments and Analytical Methods” provided by Hangzhou Lianchuan Biological Information Co., Ltd.

### 2.3 Prediction of differential metabolite targets

Three-dimensional structure and SMILES string of differential metabolite were obtained from PubChem database. The possible targets of differential metabolite were obtained from two databases: Swiss Target Prediction database, STITCH database, under the selection conditions that “Homo sapiens” was selected as the species and the possibility was “> 0.1”. All the targets obtained from the two databases were put together, and then the reduplicated targets were deleted to obtain differential metabolite binding targets.

### 2.4 Identification of genes associated with prostate cancer

The word “Prostate cancer” was used as keywords to search for prostate cancer -related targets in three databases: Therapeutic Target Database (TTD), GeneCards database and Online Mendelian Inheritance in Man (OMIM). The targets obtained from the three databases were put together, and then the reduplicated and irrelevant targets were deleted to obtain prostate cancer specific targets.

### 2.5 Enrichment analyses of gene ontology and kyoto encyclopedia of genes and genomes pathway

The thirty-two overlapping targets of six metabolic markers and Prostate cancer were obtained for enrichment analyses of GO terms (biological process, cellular component and molecular function) and KEGG pathways. GO terms enrichment analysis is a method of functional annotation and enrichment analysis of the studied genes based on the GO database. It calculates the enrichment degree and significance of the studied genes in each GO term through statistical algorithms. KEGG pathways enrichment analysis is a bioinformatics method used to understand the function of the studied genes and the signaling pathways in which they are involved. The GO terms and KEGG pathways with *P* < 0.05 were used for enrichment analyses to identify significantly enriched GO terms and KEGG pathways. The graphs of enrichment analyses were obtained using the R package.

### 2.6 Molecular docking method

Interactions between alpha-Tocomonoenol and Androgen receptors were analyzed using the molecular docking method. 3D structure of alpha-Tocomonoenol obtained from the PubChem database, and then was saved as PDB formats. 3D structures of the Androgen receptor proteins were obtained as PDB format files from the RCSB Protein Data Bank (PDB) database. The PDB formats of the proteins and alpha-Tocomonoenol were imported to the CB-Dock2 online for predicting their binding sites and affinity. The Interaction between dihydrotestosterone and Androgen receptors was also analyzed by the same method.

### 2.7 Cell culture

Two human PCa cell lines LNCaP, 22Rv1and one human prostate epithelial cell line RWPE-1 were purchased from American Type Culture Collection (Manassas, VA). Cells were cultured in RPMI 1640 medium (PYG0006, Boster) supplemented with 10% FBS (CTCC-002-071-50, Meisen), 1% penicillin and 1% streptomycin (15140122, Gibco), and maintained at 37 °C in humidified incubator with 5% carbon dioxide. All cell lines were authenticated by short tandem repeat profiling and tested immediately for Mycoplasma upon receipt using MycoAlert Mycoplasma Detection Kit (Lonza Bioscience, Basel, Switzerland).

### 2.8 Cell viability and colony formation assays

The effect of alpha-Tocomonoenol on the proliferation of LNCaP, 22Rv1 and RWPE-1 cells was determined by CCK8 assay. The cells were seeded in 96-well plates at densities of 6 × 10^3 cells (LNCaP) or 3× 10^3 (22Rv1) per well. at the same time, the two cell lines were treated with different concentrations of alpha-Tocomonoenol for 0–72 h. Subsequently, 10 µL of CCK8 reagent was added to each well, according to the manufacturer’s instructions. After incubation at 37 °C for 1.5h, optical density was measured at 450 nm.

LNCaP and 22Rv1 cells were seeded at 3000/well into 6-well plates for colony formation. After 10 days of culture, cells were fixed with 4% paraformaldehyde for 30 min and stained with 0.1% crystal violet for 20 min at room temperature in the dark. Visible colonies (diameter > 0.1 mm) were photographed.

### 2.9 Knockdown of AR by siRNA transfection

Cells were seeded onto a 6-well plate with 40%–50% confluency at the time of transfection. 5 mL of Lipofectamine 3000 Transfection Reagent (Thermo Fisher Scientific) and 10 mL of siRNA plasmids were separately prepared and diluted in 250 mL of 1640 Medium (Gibco BRL). The two diluted solutions were then mixed and incubated at room temperature for 20 min. After incubation, the plasmid-lipid complex was added to cells for transfection. Double-stranded siRNA oligonucleotides for AR were con-structed and purchased from Tsingke Biotechnology (Beijing, China). The sequences of siRNAs are as follows: AR siRNA-1: 5’- GGUUCUCUGCUAGACGACA-3’, AR siRNA-2: 5’-GCUCUCUAGCCUCAAUGAA-3, AR siRNA-3: 5’-GCUCAAGACGCUUCUACCA-3’

### 2.10 Statistical analysis

The Statistical Package for the Social Sciences (SPSS) ver. 25 (IBM, Armonk, NY, USA). We carried out stepwise logistic regression (SLR) analysis, with the significance level of *P* < 0.05. Our cases were grouped into two groups: one (n =30) with a positive pathological result and the other (n = 30) with a negative pathological result.

SPSS was used to build our SLR model, with the *P* < 0.05. At the 8th step of variable selection, a statistically significant result was obtained (*P* < 0.001). In terms of a goodness-of-fit measure, the regression model had a R² value of 0.550, and an Adjusted R² value of 0.499.

## 3 Results

### 3.1 Characteristics of participants

**Figure 1:**
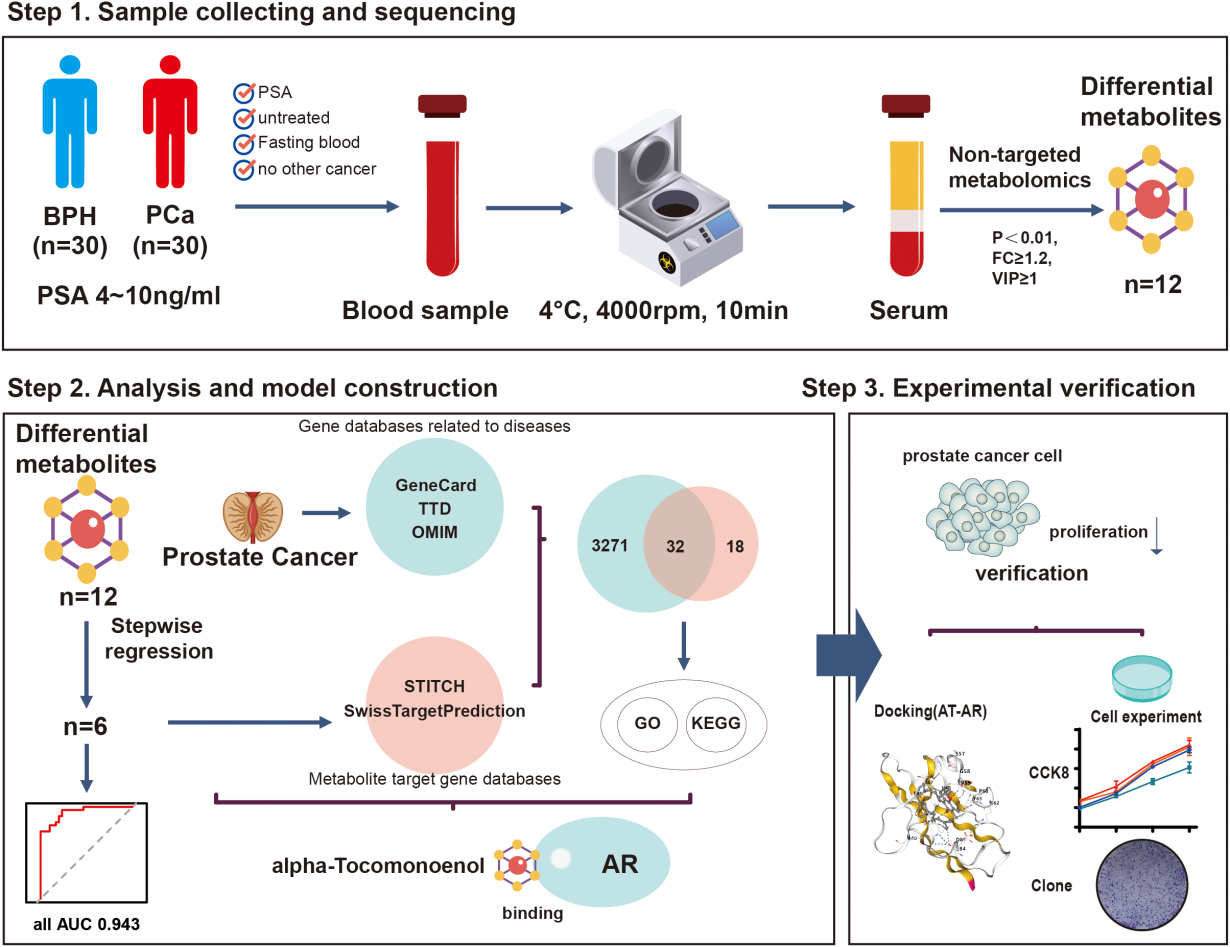
The flow chart of the present study. A total of 60 patients were included in this study. The patients were divided into the PCa group (n=30) and the BPH group (n=30) according to their pathological results. Non-targeted metabolomics analysis was performed to identify the biomarkers and construct a novel multi-omics prediction model. Through experiments, we found that A-T, one of the biomarkers, can inhibit the proliferation of prostate cancer cells by binding to AR.

Based on the pathological results and inclusion/exclusion criteria, finally, 60 patients were enrolled from the Urology Department of the Jiangnan University Medical Center from November 2023 to June 2024. All of them met our inclusion criteria, including 30 patients with PCa and 30 patients with BPH. **Figure 1** shows the overall process of study.

Characteristics of the participants are shown in **Table 1**. Gleason Score and WHO Grade were calculated in the malignant group. Although PSA levels of all patients were in the gray zone of 4-10ng/ml, the mean PSA value of the PCa group was higher than that in the BPH group (*P* = 0.001). There was no significant difference in other characteristics between the two groups.

**Table 1:** Characteristics of the participants.

	PCa group	BPH group	*P* value
Patients	30	30	–
Mean BMI	23.88	23.47	0.60
Mean Age (year)	71.93	69.30	0.24
Mean PSA (ng/l)	7.69	6.09	0.001
Gleason Score			
5	0	–	–
6	5	–	–
7	16	–	–
8	3	–	–
9	5	–	–
10	1	–	–
WHO Grade^1^			
Grade Group 1	6	–	–
Grade Group 2	9	–	–
Grade Group 3	6	–	–
Grade Group 4	3	–	–
Grade Group 5	6	–	–
Diabetes			0.77
Yes	8	7	
No	22	23	
Smoking			0.77
Yes	7	8	
No	23	22	
Drinking			0.57
Yes	8	10	
No	22	20	
Mediterranean diet			0.158
Yes	7	14	
No	16	14	
No record	7	2	
Family history of Pca			0.47
Yes	3	1	
No	20	27	
No record	7	2	
bald			0.60
Yes	9	13	
No	14	15	
No record	7	2	
Circumcision			0.82
Yes	3	2	
No	20	26	
No record	7	2	

1. WHO Classification of Tumors Grade.

### 3.2 Alterations in serum metabolites in PCa patients compared to BPH patients

**Figure 2:**
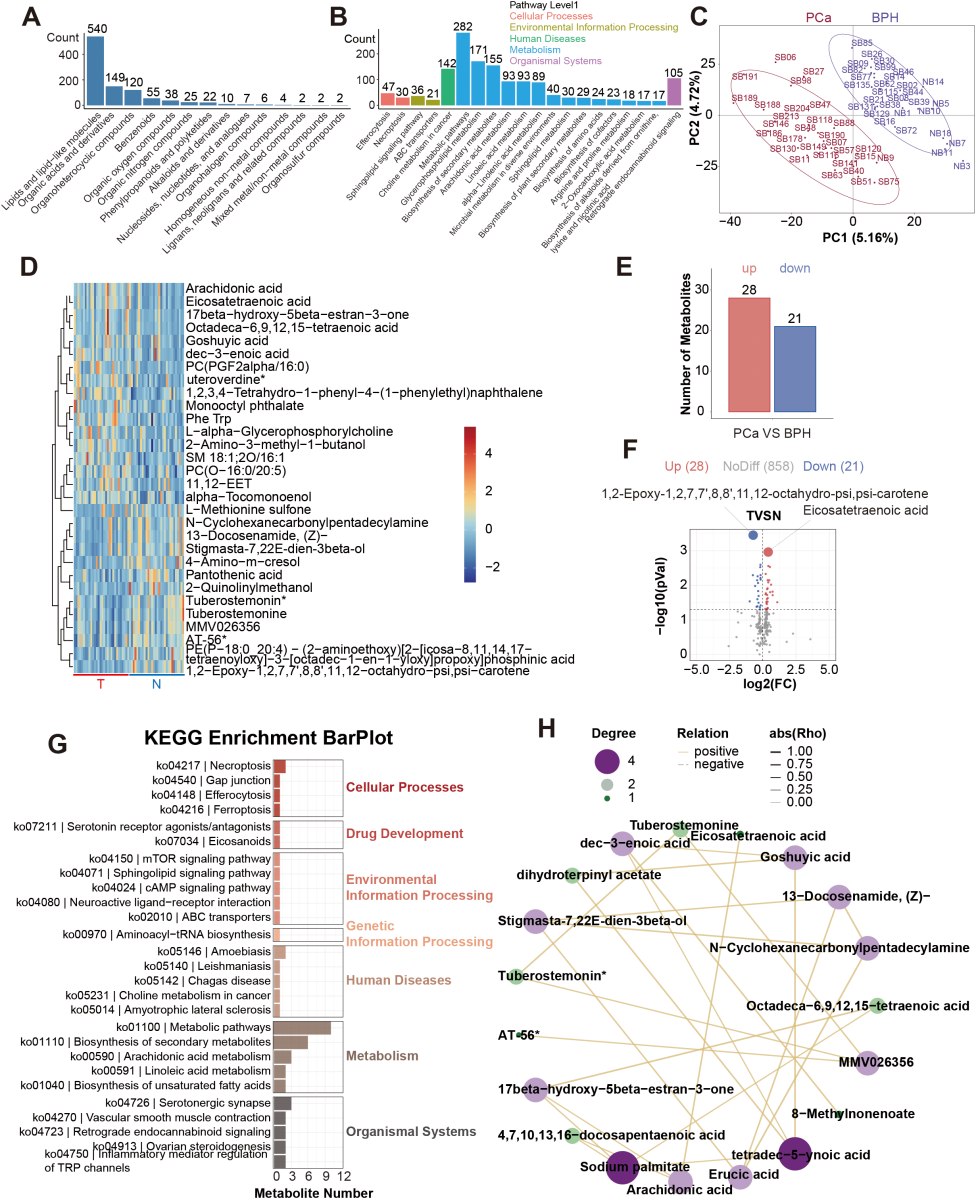
Profiling results of non-targeted metabolites. **(A)**. HMDB SuperClass Classification Annotation Statistics Chart. **(B)**. Top 20 Pathways by Metabolite Count. **(C)**. Inter-group PLS-DA Score Plot. **(D)**. Heatmap of the top 30 differential metabolites. **(E)**. Differentially serum metabolites between the PCa and BPH groups. **(F)**. Volcano plot of the differential serum metabolites. **(G)**. KEGG enrichment analysis of the differential serum metabolites. **(H)**. Correlation analysis network diagram of the top 30 differential metabolites.

Seven thousand nine hundred and seventy-three primary metabolites and 1098 secondary metabolites were identified from a total of 60 serum samples. Secondary metabolites were annotated and classified according to the Human Metabolome Database (HMDB) and the KEGG database. And we found that most of the metabolites were lipids and lipid-like molecules, and most of them are related to metabolic pathways such as glycophospholipid metabolism and choline metabolism in cancer (**Figures 2A, B**).

PLS-DA (Partial Least Squares Discriminant Analysis) was performed between the two groups (**Figure 2C**). PLS-DA score graph showed that there is a significant difference between the two groups. There was a significant difference between the two groups in PLS-DA score graph, and the permutation test graph indicated that our model was not overfitting (**Supplementary Figure 1A**). Significant results were considered to be those with *P*<0.05 and Variable Importance in Projection (VIP) score ≥ 1. Then we found 781 differential metabolic ions between the two groups (**Supplementary Figure 1B**). We used Principal Components Analysis to show the composition of serum metabolites (**Supplementary Figure 1C**). This study focused on the differential results of the secondary metabolites, which were more reliable. The heat maps of the top 30 differential metabolites were presented (**Figure 2D**). Compared with the benign group, 28 metabolites were up, and 21 metabolites were down in the malignant group. 1, 2-Epoxy-1, 2, 7, 7’, 8, 8’, 11, 12-octahydro-psi, psi-carotene and Eicosatetraenoic acid were the differential metabolites with the smallest *P* value (**Figures 2E, F**). Meanwhile, we conducted KEGG enrichment analysis on differential metabolites and constructed a correlation analysis network diagram among different metabolites (**Figures 2G, H**). The top 30 metabolite set with the smallest *P* value and FDR value in the KEGG pathway GSEA analysis results were displayed in a bar chart (**Supplementary Figure 1D**). We selected the metabolite set with Nominal *P*<0.05 to draw the Enrichment Score (ES) Line Chart. And the ES line charts of the items with *P*<0.05 were displayed (**Supplementary Figures 1E–K**).

**Figure 3:**
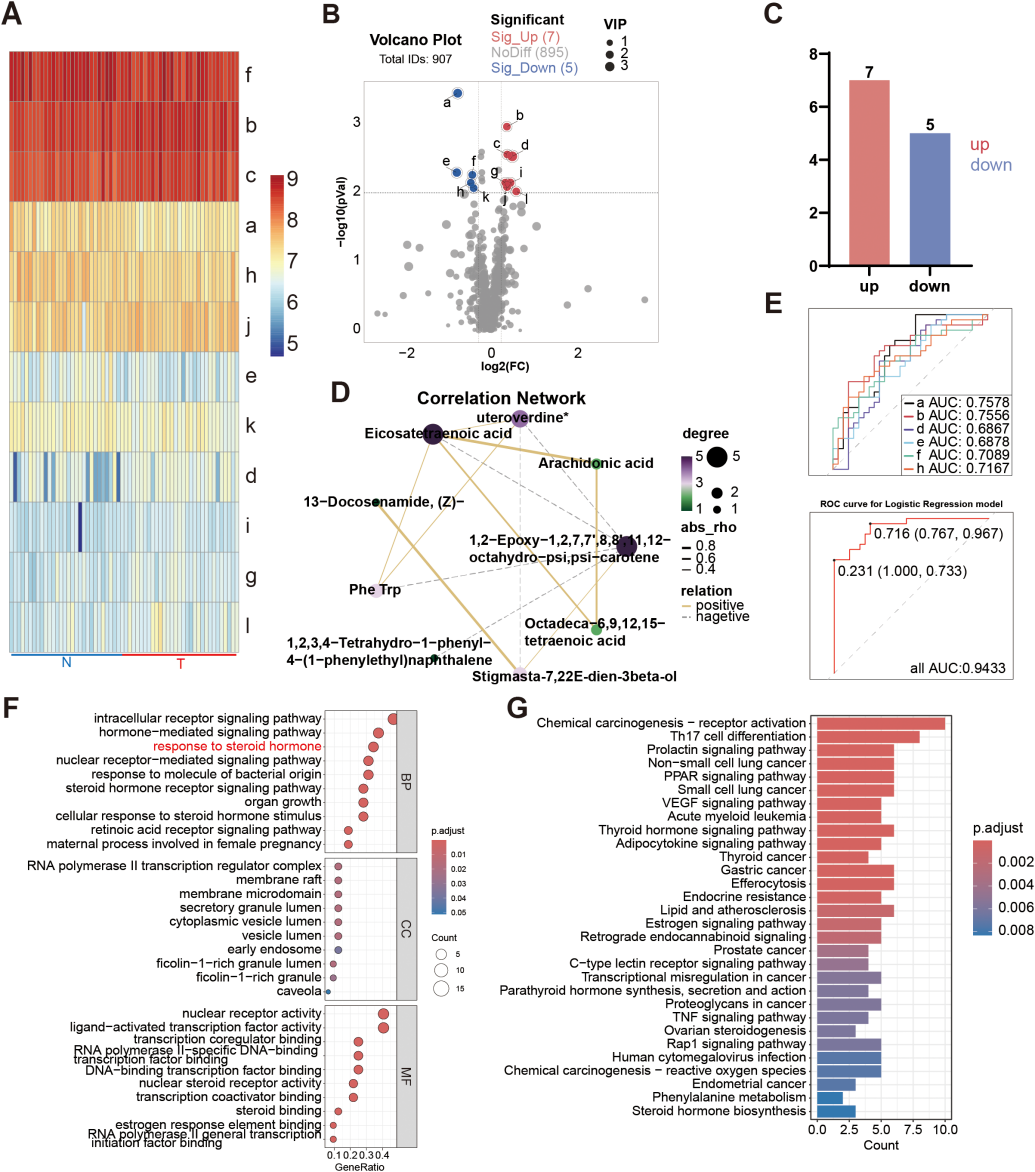
Differential serum metabolites (*P* < 0.01, VIP≥1) of patients with the PSA level in 4-10ng/ml. **(A)** Heatmap of the differential metabolites. a: 1, 2-Epoxy-1, 2, 7, 7’, 8, 8’, 11, 12-octahydro-psi, psi-carotene; b: Eicosatetraenoic acid; c: Arachidonic acid; d: alpha-Tocomonoenol; e: 2-Quinolinylmethanol; f: 13-Docosenamide, (Z)-; g: 21H-Biline-8, 12-dipropanoicacid, 3, 18-diethenyl-1, 19, 22, 24-tetrahydro-2, 7, 13, 17-tetramethyl-1, 19-dioxo-; h: 4-Amino-m-cresol; i: Phe Trp; j: 1, 2, 3, 4-Tetrahydro-1-phenyl-4-(1-phenylethyl)naphthalene; k: stigmasta-7, 22E-dien-3β-o1; l: Octadeca-6, 9, 12, 15-tetraenoic acid. **(B)** Volcano plot of the differential metabolites. **(C)** Differentially serum metabolites (*P* < 0.01, VIP≥1) between the PCa and BPH groups. **(D)** Correlation analysis network diagram of the differential metabolites. **(E)** ROC curves of the biomarkers in individual and total. a. b. d. e. f. h, AUC of a: 0.7578(95%CI: 0.6353-0.8802); AUC of b: 0.7556(95%CI: 0.6286-0.8825); AUC of d: 0.6867(95%CI: 0.5490-0.8243); AUC of e: 0.6878(95%CI: 0.5543-0.8213); AUC of f: 0.7089(95%CI: 0.5773-0.8405); AUC of h: 0.7167(95%CI: 0.5856-0.8477); AUC of total: 0.9433 (95%CI: 0.8913-0.9954). **(F)** GO enrichment analysis of the differential serum metabolites. **(G)** KEGG enrichment analysis of the differential serum metabolites.

Considering the redundancy of 38 candidate features in the diagnostic model, we implemented more stringent statistical thresholds to optimize feature selection. We adjusted the analysis criteria to *P* < 0.01, FC≥1.2, VIP≥1 for another analysis. There were 12 different serum metabolites (**Figure 3A**). Among them, 7 metabolites were up, and 5 metabolites were down in the malignant group (**Figures 3B, C**). We also constructed a correlation analysis network diagram among 12 different metabolites (**Figure 3D**).

### 3.3 Serum metabolites effectively distinguish patients with PSA in gray zone

**Figure 4:**
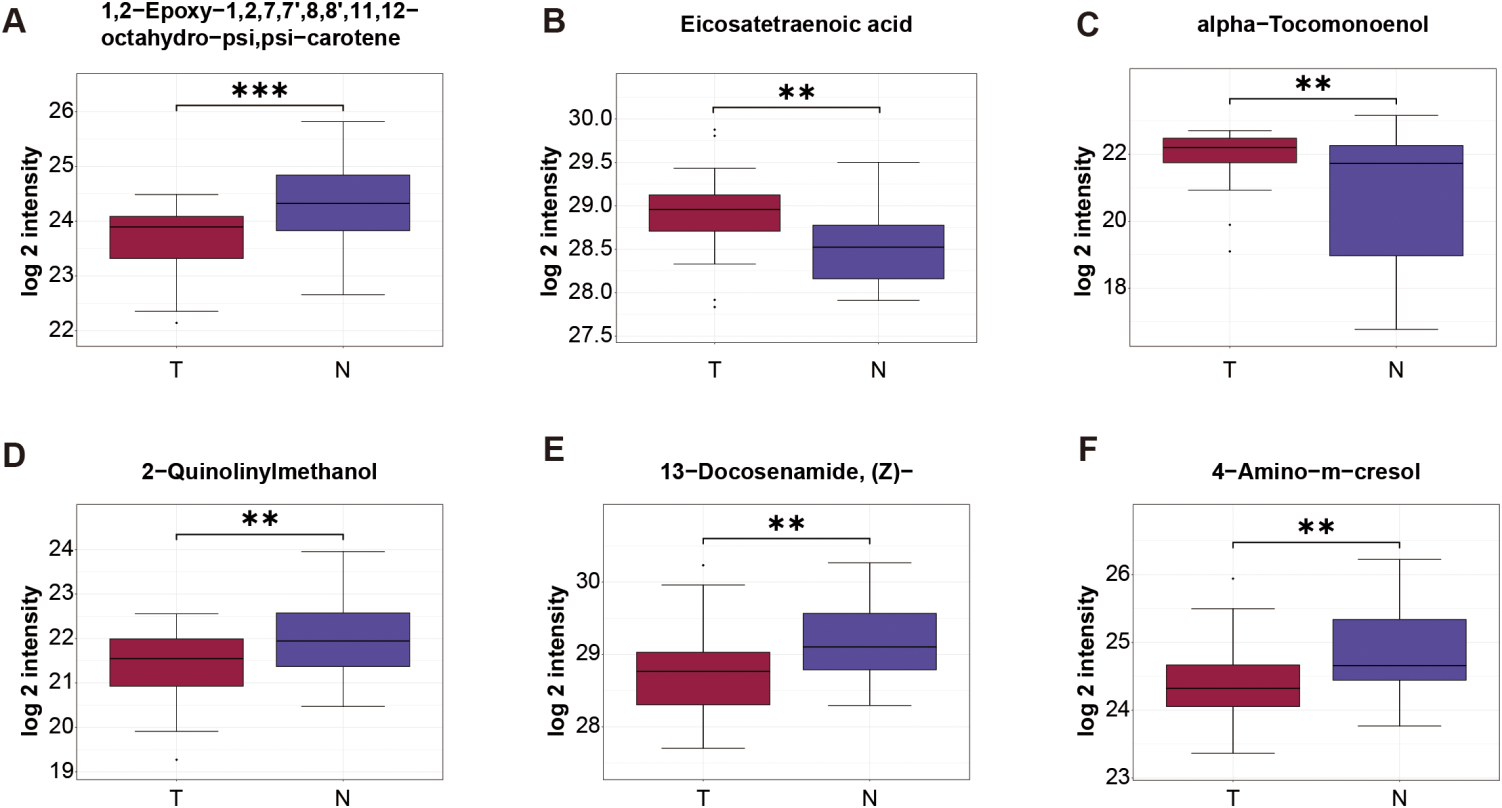
Expression level of serum metabolite obtained by stepwise regression analysis. Data are shown as mean ± SD. **P < 0.01, ***P < 0.001.

The stepwise regression was used to build a single-omics model. The result showed that 6 metabolites were selected, finally, including 1, 2-Epoxy-1, 2, 7, 7’, 8, 8’, 11, 12-octahydro-psi, psi-carotene, Eicosatetraenoic acid, alpha-Tocomonoenol, 2-Quinolinylmethanol, 13-Docosenamide, (Z)- and 4-Amino-m-cresol (Complete methodological details are available in the **Supplementary Tables S1–S5**). And we got the equation = 0.692-1.277E-8*1, 2-Epoxy-1, 2, 7, 7’, 8, 8’, 11, 12-octahydro-psi, psi-carotene-4.679E-8*2-Quinolinylmethanol-7.383E-9*4-Amino-m-cresol+1.060E-9* Eicosatetraenoic acid+4.796E-8* alpha-Tocomonoenol-3.856E-10*13-Docosenamide, (Z)-. Then we got the AUC value of these 6 metabolites, which was 0.9433 (95%CI: 0.8913-0.9954; Sensitivity:76.67; specificity:96.67; PPV: 0.9375; NPV: 0.8108) (**Figure 3E**). **Figure 4** shows the expression levels of these metabolites between the two groups.

### 3.4 Functional enrichment analysis of 32 overlapping targets

A total of 50 differential metabolites targets were identified by searching Swiss Target Prediction database, and STITCH database. A total of 3303 prostate cancer specific genes were identified by searching GeneCards database, TTD and OMIM, of which 32 genes overlapped with A-T binding targets. The detailed information of the 32 overlapping targets is listed in **Supplementary Table S6**.

Enrichment analyses of GO terms and KEGG pathways of the 32 overlapping targets of differential metabolites and prostate cancer were carried out via R. As shown in **Figure 3F** that lists detailed information on the top 10 most significantly enriched GO terms, the top 10 molecular function terms mainly involved in the nuclear receptor activity, transcription and steroid binding. The top 10 cellular component terms were mainly associated with the cell structure and function. The top 10 biological process terms mainly involved in the signaling pathway, steroid hormone.

The bar plot (**Figure 3G**) illustrates the top 20 most significantly enriched KEGG pathways with their corresponding target genes involved. A relatively larger number of target genes were involved in Chemical carcinogenesis − receptor activation, suggesting that this pathway was enriched with the most count of genes. At the same time, it was the most significantly enriched pathway.

### 3.5 A-T may inhibit the proliferation of LNCaP and 22Rv1 through AR

Given the androgen-sensitive profile of our cohort comprising exclusively early-stage prostate cancer patients, we strategically prioritized hormone-associated pathways identified through systematic GO and KEGG enrichment analyses. This focused approach revealed that metabolite A-T - a key component of our diagnostic model - demonstrates potential regulatory interplay with the androgen receptor (AR), as evidenced by the significant enrichment of AR-related signaling (GO:0048545 response to steroid hormone, adjusted *P* value: 1.9028× 10^−9^) within the molecular pathway network.

**Figure 5:**
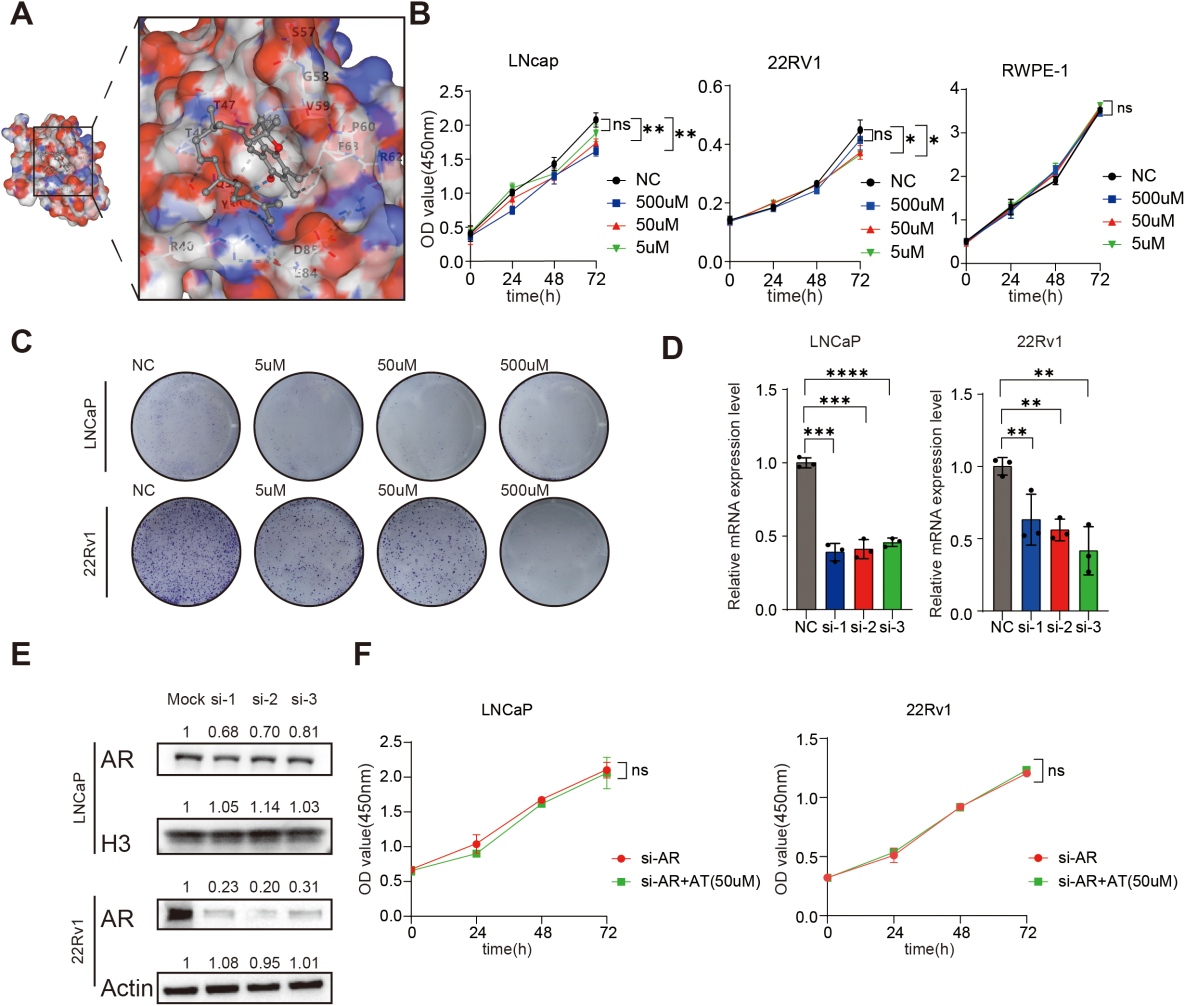
A-T inhibits LNCaP and 22Rv1 by combining AR. **(A)**. A-T can be combined with AR. **(B, C)**. A-T can inhibit the proliferation of LNCaP and 22Rv1. **(D, E)**. The AR expression of LNCaP and 22Rv1 was silenced using siRNA. **(F)**. When AR was silenced, AT failed to inhibit LNCaP and 22Rv1 proliferation. Data are shown as mean ± SD. *P < 0.05, **P < 0.01, ***P < 0.001, ****P < 0.0001.

To delineate the potential mechanisms underlying A-T/AR interactions - which may involve either direct ligand-receptor binding or indirect allosteric modulation - we conducted systematic molecular docking simulations using the CB-Dock2 platform (**Figure 5A**). It is noteworthy that AR demonstrated robust binding affinity toward A-T, with Vina scores of less than −5.0 (The bound amino acid sites are GLN38, GLN39, ARG40, SER43, ALA44, THR46, THR47, VAL48, SER57, GLY58, VAL59, PRO60, ARG62, PHE63, GLU84, ASP85, TYR89). Meanwhile, we also simulated the docking of dihydrotestosterone with AR, whose Vina score was -3.5 to -6.2. This observation suggests that the interaction between A-T and AR may be achieved through direct combination. And early-stage prostate cancer cells express AR. We speculate that the spontaneous binding of A-T with AR plays a crucial role in the molecular pathway leading to A-T-induced PCa.

To demonstrate the relationship between metabolic biomarkers and PCa, we conducted A series of *in vivo* experiments between A-T levels and two commonly used Androgen receptor-positive prostate cancer cell lines cell lines (LNCaP and 22Rv1). The results of prediction of Differential metabolite targets showed that the content of A-T was higher in the serum of PCa patients (**Figure 4C**). At the same time, we treated LNCaP and 22Rv1 with A-T for 0h to 72 h, in three different concentrations of A-T respectively, to determine the relationship between A-T and PCa. The results of CCK8 assay and Colony formation showed that the cell viability of LNCaP and 22Rv1 descended with the intervene of A-T (**Figures 5B, C**). Those results were contrary to our profiling results. In parallel, we evaluated the cytotoxicity of A–T and found that it did not affect RWPE-1 viability at the same concentration (**Figure 5B**), indicating negligible toxicity of A–T toward normal human prostate cells. This finding is consistent with previous reports (16).

To verify whether AR participated in the effect of A-T on LNCaP and 22Rv1, we conduct a combination of knockdown and drug treatment experiments (**Figures 5D, E**). We found that after silencing the AR of the LNCaP and 22Rv1, there was no statistically significant difference between the A-T dosing group and the control group. It suggests that A-T may mediate the antiproliferative effect through AR (**Figure 5F**).

## 4 Discussion

PCa is the second most common cancer in the male population and has a high incidence and mortality rate worldwide. Early detection of PCa can not only improve the survival rate and life quality of the patients, but also significantly reduce the economic and psychological burden. Liquid biopsy of PSA is the most widely used approach for PCa screening. However, PSA still has the significant disadvantage of low specificity. In particular, patients with the PSA level of 4-10ng/ml fall into a diagnostic gray zone. These patients often suffer from excessive medical treatment and are severely damaged physically and mentally. Therefore, it is both important and urgent to effectively distinguish patients with PSA gray zone level.

Metabolites play a huge role in the early diagnosis of many tumors (17, 18). Serum metabolites exhibit greater stability compared to fecal and urinary metabolites, while maintaining similarly straightforward collection procedures. For patients requiring long-term monitoring, this advantage enables convenient at-home cancer testing, significantly reducing time commitment and logistical burdens. Furthermore, the minimally invasive nature of serum sampling enhances patient acceptance, thereby improving adherence to regular surveillance protocols, which is a critical factor in effective disease management.

So untargeted metabolomic analysis was performed on serum samples from patients with PSA levels of 4–10 ng/ml. The ES line charts show some of the potential pathways by which those metabolites are involved in prostate cancer.

Arachidonic acid (AA) pathway is an important metabolic pathway which plays a key role in normal and various pathophysiological functions. For example, AA pathway metabolizing enzymes and their products can modulate tumor progression through several mechanisms, like orchestrating the inflammatory response, regulate multiple cellular processes and so on (19). Prostaglandins, leukotrienes and cyclooxygenases (COXs) are important parts of the AA pathway, and they are all closely related to the progression of prostate cancer (19, 20). The ES line plot demonstrated the upregulation of the arachidonic acid metabolism-related gene set in PCa patients, which aligns with prior research findings.

Bile acids (BAs) function not only as signaling molecules modulating multiple physiological pathways (e.g., FXR and TGR5 signaling) (21–23) but also dynamically shape gut microbiota composition (24, 25) and facilitate bacterial translocation into tissues (26, 27). Our data identified significant upregulation of bile secretion-related genes in BPH patients compared to PCa patients. Collectively, these findings suggest that BA homeostasis may exert tumor-suppressive effects through intestine and tissue microenvironment, while BA metabolic dysregulation could directly or indirectly drive prostate carcinogenesis, such as excessive inflammation, heightened oxidative DNA damage, and so on (26).

The ES line plot revealed significant upregulation of the fatty acid biosynthesis pathway in PCa patients, suggesting that enhanced fatty acid biosynthesis may drive prostate carcinogenesis and progression. Consistent with these findings, prior studies have demonstrated positive correlations between fatty acid synthase (FASN) expression/metabolite levels and PCa disease stages. Importantly, *de novo* fatty acid synthesis is critically involved in both proliferation and metastasis of PCa (28).

Interestingly, microbial metabolism was enriched in our result, demonstrating the close association between metabolites and microorganisms in Pca. The ES curve analysis showed significant downregulation of bacterial metabolism-associated gene sets in PCa patients, suggesting a potential tumor-suppressive role of the human microbiota through microbial-host metabolic crosstalk.

Our study found significant differences in the serum metabolites between BPH and PCa patients with PSA in the gray zone. At first, we took *P* < 0.05 and VIP ≥1 as the screening criteria and screened out 49 secondary differential metabolites. But excessive features in diagnostic models may lead to overfitting. Therefore, we established new screening criteria (*P* < 0.01, VIP ≥ 1) and re-analyzed the profiling data, identifying 12 secondary differential metabolites that met these thresholds.

The key serum metabolites were identified by serum metabolite profiling and stepwise regression. Specifically, the 6 metabolic biomarkers were 1, 2-Epoxy-1, 2, 7, 7’, 8, 8’, 11, 12-octahydro-psi, psi-carotene, Eicosatetraenoic acid, alpha-Tocomonoenol, 2-Quinolinylmethanol, 13-Docosenamide, (Z)- and 4-Amino-m-cresol. Although we had constructed the diagnostic model and equations composed of them, more samples were still needed to determine the CUT-OFF value.

From our profiling results, there is a significant difference in the level of A-T between the blood of PCa patients and the blood of BPH patients. Because of this characteristic, it could distinguish between PCa patients and BPH patients in the gray area of PSA. It is interesting that the results of cell experiments contradict the results of profiling. In result of our serum metabolite profiling, A-T was found to be higher in the serum of PCa patients. However, cell experiments showed that A-T could inhibit the proliferation of LNCaP and 22Rv1. Cause most of the prostate cancer samples we collected were early-stage prostate cancer, we suspected that to maintain homeostasis, the gut absorbs more vitamin E to suppress inflammation. Notably, metabolomic profiling of peripheral blood revealed significantly elevated levels of A-T in PCa patients compared to BPH patients. This observation may reflect a potential tumor-protective mechanism whereby PCa cells extrude intracellular A-T into systemic circulation, to mitigate its growth-inhibitory effects within the tumor microenvironment.

The phenomenon where metabolite levels are elevated in patients with a disease, yet the metabolites themselves exert inhibitory effects on the disease, has been documented in numerous studies. In previous research, although serum PAGln concentration was higher in PCa patients compared to the non-cancer group, experiments demonstrated that PAGln can suppress the growth and metastasis of PCa cells. lv et al. also provided evidence that the CCNG2 and Wnt/β-catenin pathways may mediate the anti-cancer effects of PAGln in PCa (29).

Furthermore, in a study by Li Ren Kong et al., methylglyoxal (MGO), a glycolytic metabolite, was found to accumulate at higher levels in pancreatic tumors. At high doses, MGO exerts cytotoxic effects on cancer cells through multiple mechanisms, including induction of cell growth arrest, activation of apoptosis/necrosis signaling, and inhibition of glycolysis (30).

Beyond metabolites, similar paradoxical phenomena are observed with RNAs and tumor suppressor genes. For instance, a study on colorectal cancer (CRC) reported that CXCL11 gene expression is upregulated in colon adenocarcinoma (COAD) tissues (31). However, patients with high CXCL11 expression exhibited longer survival, potentially due to its role in promoting anti-tumor immunity. Consequently, CXCL11 was identified as an independent prognostic biomarker in COAD patients. Similarly, while Plk1 is overexpressed in breast cancer, it can also induce chromosomal instability, interfere with mitosis, and suppress tumors (32).

A theory proposed by Chaoyi Chen et al. suggests that tumor cells may actively secrete tumor-suppressive circRNAs via exosomes to maintain their malignancy (33). This aligns with the hypothesis mentioned in our study, whereby prostate cells may expel A-T extracellularly through a specific self-protective mechanism, leading to elevated peripheral blood levels of A-T. However, this theory remains speculative and requires substantial experimental validation.

Our study has several limitations that warrant consideration. The diagnostic model was developed with a limited sample size and absence of an independent validation cohort, necessitating prospective, multicenter studies to establish more clinically relevant CUT-OFF values. Furthermore, while the A-T/AR interaction was predicted through computational analyses, the mechanistic investigation remains incomplete without experimental validation via site-directed mutagenesis experiments targeting specific amino acid residues. Nevertheless, this investigation represents a conceptual advancement as the first demonstration of untargeted metabolomics’ novel utility in addressing diagnostic challenges within the PSA gray zone (4-10ng/ml) - a clinically critical yet diagnostic ambiguous parameter in prostate cancer screening.

Based on serum metabolomics, this study identified six biomarkers for assisting in the diagnosis of patients with PCa and BPH in the PSA grey area and utilized them to construct a diagnostic model. Meanwhile, we discovered that A-T might inhibit the proliferation of prostate cancer cells by binding to AR. These findings may contribute to the early diagnosis and treatment of prostate cancer.

## Data Availability

The original contributions presented in the study are publicly available. This data can be found here: https://ngdc.cncb.ac.cn/omix/; OMIX012859-01.
