# Supplementary material for: Serum metabolites with diagnostic potential in prostate cancer and the inhibitory effects of alpha-Tocomonoenol on prostate cancer cells
Source: Front Oncol. 2025 Nov 20;15:1691767. doi: 10.3389/fonc.2025.1691767 (PMC12675167; doi:10.3389/fonc.2025.1691767)
Supplement: Supplementary file 1 [file DataSheet1.docx]

**Serum metabolites with diagnostic potential in prostate cancer and the inhibitory effects of alpha-Tocomonoenol on prostate cancer cells**

**
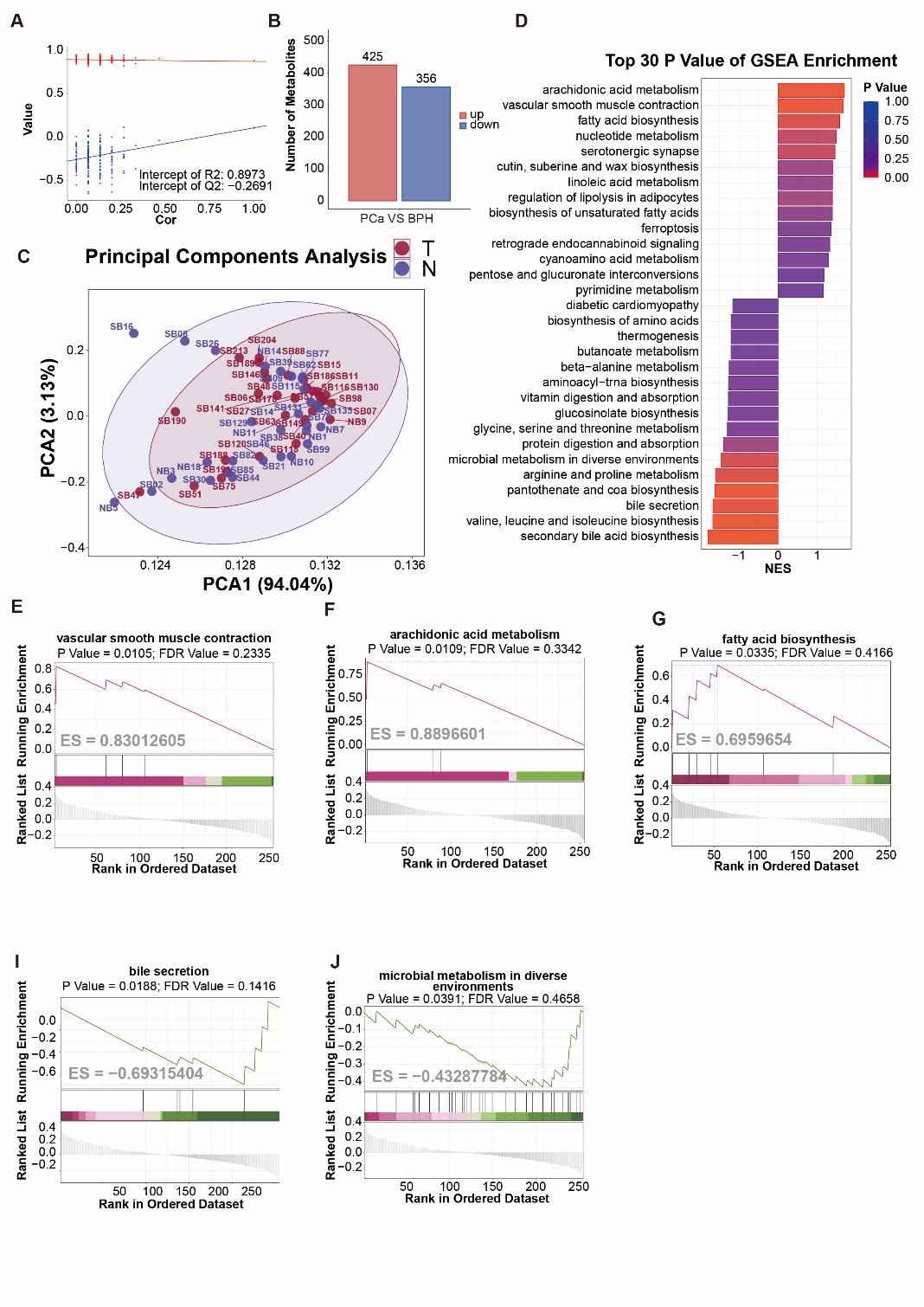
**

**Supplementary Figure 1. Results of untargeted metabolomics sequencing of the patients with the PSA level in 4-10ng/ml.**

**(A)**. Permutation test diagram for all samples.

**(B)**. Differential metabolite ions between PCa and BPH groups.

**(C)**. Principal Components Analysis between PCa and BPH groups.

**(D)**. GSEA analysis of the differential serum metabolites with the top 30 ***P*** values.

**(E-J)**. Results of the GSEA analysis regarding differential metabolites.
